# Book Reviews

**Published:** 1903-08

**Authors:** 


### *BOOK REVIEWS.*

THE PREVENTION OF DISEASE. Translated from the German, with an Introduction by H. Timbrell Bulstrode, M.A., M.D., Cantab, D.P.H. In two volumes. Funk and Wagnalls Co., 30 Lafayette Place, New York, 1903.

This is the most comprehensive work on the subject of preventive medicine that has yet appeared. The general considerations of the subject are first taken up, then the various physiologic systems and organs are discussed in detail in regard to prophylaxis.

Among other names more or less notable of German authors, is that of the American, Max Einborn, whose work in connection with diseases of the stomach is so well known.

As preventive medicine is the medicine of the future, a work of this character is especially timely and acceptable, and coming as it does from sources of unquestioned authority will be read with profitable interest.

A MANUAL OF PRACTICAL HYGIENE. For students, physicians and medical officers. By Chas. Harrington, M.D. Lea Bros. & Co., Philadelphia and New York.

The second edition of this book comes revised and very much enlarged. It deals in a very direct and practical manner with the various branches of hygiene and sanitary science. The large amount of highly interesting and useful work done in connection with relation of insects to the origin and spread of disease has been condensed in a separate chapter.

We can cordially recommend the book as one of the very best of its kind.

